# Premature MicroRNA-Based Therapeutic: A “One-Two Punch” against Cancers

**DOI:** 10.3390/cancers12123831

**Published:** 2020-12-18

**Authors:** Luyue Chen, Kai Huang, Kaikai Yi, Yanlin Huang, Xinhua Tian, Chunsheng Kang

**Affiliations:** 1 Department of Neurosurgery, Zhongshan Hospital Xiamen University, Xiamen 361004, China; chenluyue@xmzsh.com (L.C.); hyl@xmzsh.com (Y.H.); 2 Department of Neurosurgery, The Second Affiliated Hospital of Nanchang University, Nanchang 330000, China; kaihuang@ncu.edu.cn; 3 Laboratory of Neuro-Oncology, Key Laboratory of Neurotrauma, Variation, and Regeneration, Ministry of Education and Tianjin Municipal Government, Department of Neurosurgery, Tianjin Neurological Institute, Tianjin Medical University General Hospital, Tianjin 300052, China; yikaikai93@163.com

**Keywords:** miRNA*, post-transcriptional regulation, premature miRNA intervention, pre-miRNA annotation, therapeutic potential

## Abstract

**Simple Summary:**

The current understanding of miRNA biology is greatly derived from studies on the guide strands and the passenger strands, also called miRNAs*, which are considered as carriers with no sense for long periods. As such, various studies alter the expression of guide strands by manipulating the expression of their primary transcripts or precursors, both of which are premature miRNAs. In this situation, the regulatory miRNA* species may interfere with the phenotypic interpretation against the target miRNA. However, such methods could manipulate the expression of two functionally synergistic miRNAs of the same precursor, leading to therapeutic potential against various diseases, including cancers. Premature miRNAs represent an underappreciated target reservoir and provide molecular targets for “one-two punch” against cancers. Examples of targetable miRNA precursors and available targeting strategies are provided in this review.

**Abstract:**

Up-to-date knowledge regarding the biogenesis and functioning of microRNAs (miRNAs) has provided a much more comprehensive and concrete view of miRNA biology than anyone ever expected. Diverse genetic origins and biogenesis pathways leading to functional miRNAs converge on the synthesis of ≈21-nucleotide RNA duplex, almost all of which are processed from long premature sequences in a *DICER*- and/or *DROSHA*-dependent manner. Formerly, it was assumed that one mature strand of the duplex is preferentially selected for entry into the silencing complex, and the paired passenger strands (miRNA*) are subjected to degradation. However, given the consolidated evidence of substantial regulatory activity of miRNA* species, currently, this preconception has been overturned. Here, we see the caveat and opportunity toward exogenously manipulating the expression of premature miRNA, leading to simultaneous upregulation or downregulation of dual regulatory strands due to altered expressions. The caveat is the overlooked miRNA* interference while manipulating the expression of a target miRNA at the premature stage, wherein lies the opportunity. If the dual strands of a pre-miRNA function synergistically, the overlooked miRNA* interference may inversely optimize the therapeutic performance. Insightfully, targeting the premature miRNAs may serve as the “one-two punch” against diseases, especially cancers, and this has been discussed in detail in this review.

## 1. Introduction

MicroRNA (miRNA) is a class of small nonprotein-coding RNAs (ncRNAs) that are 15–27-nucleotides (nt) in length, are ubiquitously expressed, and interact with most of the mammalian messenger RNAs (mRNAs) [1]. The interplay between miRNAs and mRNAs leads to decreased translational efficiency and/or mRNA levels, which is the predominant reason behind decreased protein production [2,3,4]. It is estimated that over 60% of mRNAs are conserved targets for endogenous miRNAs, and more mRNAs will be added to this conservative estimation when less conserved sites will be taken into account, indicating the existence of a previously unexpected post-transcriptional RNA signaling network [1]. Extensive screening for functional miRNAs has become easier due to the refinement of the principles that allow the manipulation of miRNA expression; functional miRNAs will gradually overturn the central dogma of molecular biology [5]—evidence regarding involvement of miRNAs in post-transcriptional regulation is mounting, and they are considered as the key components in the maintenance of intracellular homeostasis of many physiological processes [6,7]. Apart from their intracellular functions, miRNAs are also highlighted as unpredictable, important intercellular communicators, which help in the maintenance of a homeostatic extracellular microenvironment [8]. Dysregulated miRNAs in cell type-specific or cell-state-specific gene expression patterns cause pathophysiological transitions toward neurological [9], cardiovascular [10], metabolic [11], autoimmune [12], and carcinogenic disorders [13], and in turn, provide novel diagnostic, therapeutic, and prognostic opportunities in deciphering diseases [14].

## 2. Evolving Knowledge of miRNA Biogenesis

For more than two decades, researchers have considered miRNAs as the “top star” across all classes of noncoding RNAs, which were previously considered as the genomic “dark matter.” The first miRNA, lin-4, was discovered in *Caenorhabditis elegans* in 1993 [15,16], and in 2001, these unique small RNAs were formally termed as “microRNAs” [17,18]. Since then, the miRNAome has attracted great attention due to the unknown mode of action and biosynthesis machineries of miRNAs.

**Figure 1:**
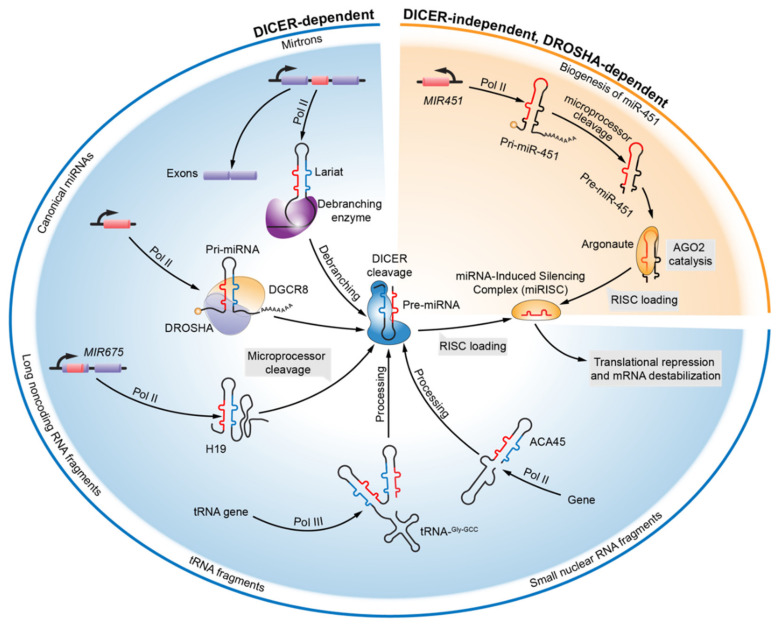
An overview of canonical and noncanonical miRNA biogenesis pathways. MiRNA genes are transcribed as primary transcripts (pri-miRNAs) by RNA polymerase II (Pol II) from intergenic or intronic regions. Pri-miRNAs are subsequently cleaved by an endonucleolytic RNase III, *DROSHA*-*DGCR8* complex, into the hairpin-structured ≈70-nt miRNA precursors (pre-miRNAs) inside the nucleus. Cytoplasm-located RNase III *DICER* formed anther complex with *TRBP*, which takes over the pre-miRNAs processing after nuclear exportation, and produces an asymmetric ≈21-nt mature miRNA duplex. The miRNA duplex is loaded into Argonaute (AGO), the core protein of miRNA-induced silencing complex (miRISC), and after target recognition mediated by seed region complementarity of the guide strand, the sequelae of complementary mRNA are translational repressed and destabilized. The “mirtron” pathway is the best characterized noncanonical miRNA biogenesis pathway. Such primary intronic transcripts form a lariat structure and require the incorporation of the lariat debranching enzyme. After debranching, the mirtrons can be sliced into hairpin pre-miRNAs in a *DROSHA*-independent manner. Besides, some other noncoding RNA species are confirmed to generate hairpin-like fragments similar to pre-miRNAs by unknown mechanisms and in this figure, long noncoding RNA H19, transfer RNA tRNA-Gly-GCC and small nucleolar RNAs (snoRNA) ACA45 are illustrated as examples. The maturation of the majority of noncanonical miRNAs require *DICER*-processing with the only exception of mature miR-451. *DROSHA* cleavage of miR-144/451 cluster produces a ≈42-nt hairpin pre-miR-451 with an unusually short stem region. Unable to be recognized by *DICER*, pre-miR-451 is further cleaved by AGO2, which frees mature miR-451. RISC, RNA-induced silencing complex; *DGCR8*, DiGeorge Syndrome Critical Region 8.

In animals, canonical miRNAs can be transcribed from either intergenic or intronic regions by RNA polymerase II (Pol II) in the nucleus, and the nascent noncoding products are the primary stem loop-containing miRNA transcripts (pri-miRNA) (Figure 1) [19]. The transformation from primary transcripts to mature miRNAs in the canonical maturation pathways require two critical cleaving processes, which are mediated by endonucleolytic RNase III proteins—*DROSHA* [20] and *DICER* [21]. During the initial processing, core of the microprocessor complex, a trimeric complex containing one *DROSHA* endonuclease and two partner protein DiGeorge Syndrome Critical Region 8 (*DGCR8*), recognizes and cleaves the primary transcripts of miRNAs, and releases the hairpin-structured ≈70-nt-long miRNA precursors (pre-miRNAs) with 2-nt-long overhangs at the 3’ end [20,22,23]. Following *DROSHA* processing, the nuclear export of the pre-miRNAs, which is dependent on Exportin-5 and its cofactor Ran-GTP, is considered critical for miRNA maturation, as the subsequent RNA processing is directed by cytoplasmic *DICER* [24]. In the cytoplasm, *DICER* takes over pre-miRNA processing; its 5’ and 3’ pocket motif binds to the 5’ and 3’ terminus of the substrate pre-miRNA [25]. In complex with Transactivation Response RNA Binding Protein (*TRBP*) or its paralogue, *DICER* cuts both the strands of pre-miRNA near the loop to form an asymmetric ≈21-nt-long mature miRNA duplex with a 2-nt-long overhang on both ends [21,26]. Once formed, the miRNA duplexes are loaded into the Argonaute (AGO) proteins, the core of miRNA-Induced Silencing Complex (miRISC), where the unwinding of the duplexes and the selection of functional strands occur, which is mediated by the Hsc70/Hsp90 chaperone machinery in an ATP-dependent manner [27]. The ATP-dependent conformational opening of AGO proteins allows the ends of the miRNA duplex to wedge into the functional N domain, and mismatches of the duplex in the seed or the guide strand at positions 12–15 drive the disassociation of the passenger strand and the maturation of miRISC, which is ready for target recognition. Unlike AGO loading, duplex unwinding is a passive procedure that does not require ATP or slicer activity [28,29]. Removal of the passenger strand facilitates the conserved Watson–Crick base-pairing between miRNA “seed” (5’ region of the miRNA centered on nucleotides 2–7) and sequences in the 3’-Untranslated Region (UTR) of the target mRNA, and the seed pairing is productively augmented by 3’ pairing with the mRNA that is optimally centered on the miRNA nucleotides 13–16 (3’-supplementary site) [30]. Upon miRNA binding, the sequelae of the complementary mRNA are translationally repressed and the mRNA is destabilized, rendering exclusive control over post-transcriptional regulation to the miRNA species [5].

A majority of the currently annotated miRNAs are canonical miRNAs, whose maturation relies on a stepwise *DROSHA*-*DGCR8* and *DICER*-*TRBP* processing. Alternative species of mature miRNA-like RNA fragments (noncanonical miRNAs) have also been identified from diverse genetic origins and biosynthetic pathways [31,32]. Noncanonical miRNAs can originate from non-miRNA genes and are produced through *DICER*- and/or *DROSHA*-independent biogenesis pathways (Figure 1). The most prevalent alternative pathway involved in the biogenesis of noncanonical miRNAs across species is the “mirtron” pathway; mirtrons are miRNA-harboring introns that produce intronic hairpin pre-miRNAs through sequential splicing and debranching [33,34,35]. In Drosophila, mutation analysis at the splicing sites of the mirtrons, particularly miR-1003, pointed out that the production of primary intronic transcripts occurs in a splicing-dependent manner, as splicing site mutations, instead of *DROSHA* knockdown, significantly reduced the accumulation of intronic pre-miRNA. Furthermore, knockdown of the lariat debranching enzyme led to a collective decrease in the intronic precursors and mature miRNAs, which confirms the presence of lariat-structured spliced introns [34,35]. In addition, several unconventional mirtrons harbor a 5’- or a 3’-tail that requires additional ribonucleolytic removal to release the terminal intronic pre-miRNA mimics, which subsequently enter the canonical biogenesis machinery of miRNA [31,36]. In brief, the production of mirtron miRNA requires splicing, debranching and *DICER* cleavage, and is *DROSHA*-independent, which is one of the unique pathways for biogenesis of noncanonical miRNAs. Moreover, there are sporadic reports about the implications of long intergenic noncoding RNAs (lincRNAs) [37], transfer RNAs (tRNAs) [38], and small nuclear RNAs (snoRNAs) [39] on the *DICER* processing pathway (Figure 1). The feature of exonic derivation distinguishes lincRNA H19-derived miR-675 from the canonical miRNAs processed through *DROSHA*- and *DICER*-machineries. It has been reported that a primary ≈2.5-kilobase (kb) Pol II-dependent transcript of H19 is cleaved by *DROSHA*-*DGCR8* complex to produce a ≈57-nt-long pre-miR-675, and the pre-miR-675 is diced by *DICER* to produce mature miR-675-3p, which subsequently represses the growth-promoting insulin-like growth factor 1 receptor (*IGF1R*) through its two 3’-UTR binding sites [37,40]. CU1276, a 22-nt-long long tRNA fragment, and the genomic origin of this miRNA-sized fragment has been mapped into the 3’ terminus of at least five annotated human tRNAs (tRNA-Gly-GCC). Positive AGO pulldown of CU1276 indicated RISC incorporation and putative binding on 3’-UTR of Replication Protein A1 (*RPA1*), which provided the molecular basis of CU1276-mediated repression of the molecular response to DNA damage in lymphomas. The processing of parental tRNA to CU1276 is *DICER*-dependent, but the structural entity responsible for *DICER* recognition remains unknown [38]. ACA45 is a typical example of a snoRNA with miRNA-like function. ACA45 can be trimmed into a smaller RNA of 20–22-nt-long in length that interacts with human AGO proteins and 3’-UTR of Cell Division Cycle 2-Like 6 (*CDC2L6*). The processing of ACA45 is independent of microprocessors but requires *DICER*. Interestingly, ACA45 is not merely a parental intermediate but also, a functional snoRNA, which possesses dual activities in post-transcriptional regulation [39]. *DICER* cleavage was considered a “must-go” road for miRNA maturation until the discovery of *DICER*-independent and AGO2 catalytic pre-miR-451 maturation in vertebrates [41,42,43]. Liberation of miR-451 precursor from the primary transcript of miR-144/451 cluster requires *DROSHA* cleaving, and the ≈42-nt-long hairpin pre-miR-451 presents with an unusually short stem region (17-nt). Mature miR-451 occupies the entire loop and a partial proximal complementary strand. AGO2 cleavage helps in freeing mature miR-451 from its precursor, while *DICER* recognition and processing is limited by the unusual stem loop [42,43]. Given that the lingering increase in the number of annotated noncanonical miRNAs does not match the emerging diversity of alternative miRNA biogenesis pathways, it is believed that only the tip of the iceberg of microRNAome has been unveiled thus far.

## 3. Expanding miRNA–RNA Regulatory Networks

Initially, miRNAs were regarded as active regulators during miRNA–mRNA interactions, whereas it was thought that the target mRNAs passively received signals for translational repression. However, the hypothesis of “competing endogenous RNAs (ceRNAs)” undermines the proactivity of regulatory miRNAs during the post-transcriptional RNA network and provides a rationale for the independent regulatory roles of protein-coding target mRNAs, whose regulating mechanisms are implemented by competitively sponging a common group of functioning miRNAs [44,45,46]. Moreover, as the world of RNA species has expanded dramatically, several novel classes of noncoding RNAs have been harnessed as competitive endogenous RNAs [47]. These have been incorporated into the miRNA–RNA interacting networks and bestowed with components for epigenetic modulations, including pseudogenes [48,49], lincRNAs [50,51,52], Transcribed Ultraconserved Regions (T-UCRs) [53,54], and circular RNAs (circRNAs) (Table 1) [55,56]. Pseudogenes are generally used to define defective genomic sequences that are similar to other functional genes [57]. *PTENP1*, a pseudogene of tumor suppressor phosphatase and tensin homolog (*PTEN*), is selectively deleted in human cancer cells, and its 3’-UTR displays tumor-suppressing activity by sponging PTEN-targeting miRNAs, which restore the antitumor activity of PTEN [48]. *TUSC2P* is a pseudogene with 89% homology to Tumor Suppressor Candidate-2 (*TUSC2*) 3′-UTR; forced *TUSC2P* expression arrests the function of common miRNAs against *TUSC2* [49]. Elucidation of the interplay between pseudogenes and miRNAs will help address the aberrant expression of pseudogenes in diseases. LincRNAs are ncRNAs >200-nt in length with complex post-transcriptional regulatory functions, including epigenetic regulations, splicing, and translational controls [58]. In the presence of HuR, lincRNA-p21 becomes unstable through the recruitment of let-7-RISC complex, and gets disassociated from Catenin Beta 1 (*CTNNB1*) and *JUNB* mRNAs, which promotes translation [50]. Acting like a ceRNA, linc-RoR shares the same regulatory miRNA program with core pluripotent transcriptional factors in the embryonic stem cells [51]. It is particularly interesting that in pancreatic cancer cells, a risk variant of linc00673 provides a mutant binding site for miR-1231, which in turn provides a novel and insightful mechanism for previously indecipherable mutations in lincRNA [52]. T-UCRs are completely conserved genetic elements that are 100% identical without any insertions or deletions between orthologous regions of human, rat, and mouse genomes [59]. However, little is known about the mechanism and consequence of dysregulated T-UCRs. A signature of five T-UCRs, *uc.269A*, *uc.160*, *uc.215*, *uc.346A*, and *uc.348*, was identified with prognostic prediction values in human leukemia. Without protein-coding abilities, the biological function of the T-UCRs is exerted by interaction with the miRNAs [53]. Another T-UCR *uc.339* was found upregulated in lung cancer, acting as a decoy for miR-339-3p, miR-663b-3p, and miR-95-5p [54]. CircRNAs comprise a novel class of ncRNAs that are processed during mRNA precursor splicing, mostly by exon back-splicing circularization [60]. The first study regarding the function of a naturally expressed circRNAs was inspired by the conceptual ceRNA theory. A brain-enriched circRNA harbors more than 70 selectively conserved miRNA binding sites and acts as a miR-7 sponge, which is termed as *ciRS-7* (circular RNA sponge for miR-7). In the same study, another miRNA sponge-like circRNA, called Sex-Determining Region Y (*Sry*), was validated as a decoy for miR-138, suggesting that the sponge effect is a general phenomenon in circRNA biology [55]. In a study, precipitation of Cerebellar Degeneration-Related Protein 1 Transcript (*CDR1as*), which was densely bound by AGO, was shown; this supported the interaction between miRISC and circRNA. Surprisingly, sequence analysis of *CDR1as* identified 74 miR-7 seed matches, which outnumbers the number of artificial sponge constructs [56]. The miRNA–RNA interacting network has expanded diversely, but it is possible that our profiling of the regulatory network is only in the budding stage and is far from thriving.

## 4. Regulatory Potential of miRNA* Species

The dynamic microRNAome—composed of miRNAs—has grown rapidly [61]. With respect to Homo sapiens, 1917 precursors and 2654 mature miRNAs have been registered (miRbase, release 22.1). Previously, it was considered that the dual strands were mature single-stranded product of the miRNA precursor, while the passenger strand (also called star strand; miRNA*) degraded quickly after loading of the guide strand, resulting in lower abundance of the star strand in the mature miRNA pool. Notably, unlike the passenger strand of the siRNA duplex, whose cleavage facilitates the functional strand into AGO2-RISC assembly, multiple mismatches of the miRNA duplex presumably inhibit the guide-strand cleavage-assisted duplex separation, resulting in the accumulation of cytoplasmic single-stranded miRNAs [62]. However, the mechanisms that assist in strand selection, dominant expression, and substantial function of the guide strand are not fully understood, and especially, several miRNA* species are demonstrated to accumulate dominantly and function post-transcriptionally in different eukaryotic species [63,64,65,66]. The molecular basis of mature miRNA strand selection and RISC-loading for repression are associated with two intrinsic rules of miRNA, 5’-nt identity and thermodynamic stability of the miRNA duplex [67]. The study of miRNA strand selection was inspired by optimization of artificial siRNA duplex to reduce “off-target” effect in siRNA-mediated gene silencing. The functional asymmetry created by the siRNA duplex is attributed to the first four base pairs and an initial G:U wobble pair, and this rule helps in predicting the dominant strand of miRNA. This study also speculated that when both strands do not contain structural attributes for functionally asymmetric RISC loading, both mature miRNAs are generated in the same amount as that of pre-miR-10 [68]. Thermodynamic profiling of pre-miRNA revealed that enhanced flexibility and an overall low internal stability profile are conserved features for the 5’ end of the guide or antisense strand miRNA of hairpin precursors [69]. Owing to the analysis of high throughput sequencing data, a U-bias and excessive purines (A and G) at the 5’ terminus of the highly expressed strands as well as a C-bias and excessive pyrimidines (U and C) at the 5’ terminus of the low expressed strands are the sequence characteristics for the strand selection of miRNAs among mammalian tissues [70]. The discovery of tissue-specific miRNA* domination indicates that the above-mentioned intrinsic rules are not stringently and ubiquitously followed, and some other intrinsic or extrinsic factors may interfere with the selection of the dominant strand. In a previous study, the accumulated miRNA* was demonstrated to be functional for the first time, and a novel theory, “target-two-sets-of-genes-with-one-pre-miRNA”, was proposed [63]. Moreover, two systemic studies reevaluated the binding of the miRNA* species by AGO proteins; they performed experimental validation of miRNA*-mediated target repression and declared that the miRNA* species pose a substantial regulatory activity within their conserved targets [65,66]. This general notion has been consolidated by many functional annotation experiments focusing on the regulatory activity of an individual miRNA*, which has not yet been annotated as a post-transcriptional modulator. Details of regulatory miRNA* species are presented in the following sections (Table 2). Triggered by the growing functional relevance of miRNA* species, the unbiased miR-5p/3p annotation of sequences derived from the 5’ and 3’ arms of the hairpin precursor is recommended to replace the utilization of miR/miR* in nomenclature, which was previously used to annotate differently expressed mature strands [71]. Increasing information about the regulatory activity of miRNA* expands the world of post-transcriptional networks and contributes to our understanding of the complexity of the dysregulated pathways in different diseases.

## 5. Caveat and Opportunity over Premature miRNA Perturbance

The establishment of biological implications of a certain miRNA requires significant perturbance of miRNA biogenesis or functioning machinery. In this review, special attention is drawn to premature miRNA perturbance where caveat and opportunity coexists. Premature miRNA perturbance commonly refers to deliberate interposition of miRNA biogenesis machinery to prevent maturation of a selected miRNA, while mature miRNA manipulation generally perturbs functioning machinery. Owing to the advances in functional miRNA asymmetric theory and miRNA-targeting oligonucleotide chemistry, commercially available synthetic oligonucleotides have gained popularity in mature miRNA interventions with high selectivity and “low off-target” activity [90,91]. Only a few studies have sought to exogenously interfere with the generation of primary or secondary miRNA transcripts so as to manipulate the expression levels of selected mature products, especially during the first decade of miRNA decoding [92,93,94,95,96]. If we retrospectively review these studies of miRNA-target validations, the vast majority of miRNAs that had undergone functional validation were guide strands (miRNA), and only few passenger or star strands were selected as the functional candidates. One of the main reasons behind this phenomenon is that the less abundant miRNAs* have not been universally recognized as a part of the post-transcriptional regulatory network. Accordingly, a caveat was filed against this premature miRNA-targeting strategy because this methodology violates the rule that most miRNA precursors produce two mature regulatory miRNAs. The maturation of miRNA is a stepwise procedure that begins from the primary transcript to the hairpin precursor and finally transforms into the mature functional form, mostly giving rise to two distinct regulatory single-stranded RNAs. MiRNA* species are currently an important part of the RNA regulatory network, regardless of their small signal to noise ratios. Tampering with the premature miRNA could manipulate the expression of target miRNA successfully, but at the same time introduce stochastically phenotypic biases through altered miRNA* expression, which is an oversight limited by contemporarily inadequate understanding of methodological flaws. Mendell and his colleagues studied the effect of miR-34a induction on gene expression by using a retroviral vector that expressed miR-34a and flanking sequence of approximately 100 bp in length, which covered the full length of the miR-34a precursor [92]. Interestingly, another study reevaluated the data and found that a preferred enrichment of miR-34a* seeds over miR-34a seeds was stronger across the top 500 most downregulated transcripts, which was not discussed in the initial report [66]; this could be because the regulatory potential of miRNA* species in the overexpression of miR-34a with a pri-miR-34a vector, which led to coaccumulation of sister strands of pre-miR-34a, was unknown. In another study, Robert et al. reported that miR-373 can target the promoter region of E-cadherin (also called *CDH1*) and Cold Shock Domain-Containing Protein C2 (*CSDC2*), and induce gene expression. Pre-miR-373 mimics were able to induce ≈30,000 times higher miR-373 expressions than that by pre-miR-373, and cotransfection with anti-*DICER* oligonucleotides proved that gene induction required the processing of pre-miR-373 into mature functional form of miR-373. The dramatically increased expression of miR-373 by its precursor is seemingly indisputable, except for that *DICER* will process pre-miR-373 into dual regulatory strands. The influence on expression and biological implication of miR-373* was not evaluated, and 3’-UTR that contained multiple putative miR-373* binding sites were not experimentally validated [93]. Animal models with targeted genome editing reign our lost- and gain-of-function customization to investigate the role of gene-of-interest in biology and disease. Two independent studies utilized the same approach of generating genetically deficient mice to investigate the regulatory activity of miR-208 and miR-223 in cardiac disease hemopathy, respectively. Northern blot revealed that miRNA knockout resulted in complete absence of pre-miRNA and mature miRNA [94,96]. Though not investigated, yet it is predictable that the result of miRNA* transcript detections should be null as well, which is an unappreciated bias in phenotypic characterization in miRNA mutant models. From our point of view, interfering with the abundance of a certain miRNA strand without influencing the complementary strand is hard to achieve by genome editing because miRNA and miRNA* are situated at close proximity to each other in the genome and are transcribed as a whole into a miRNA precursor; both strand maturation and function are highly dependent on the sequence identity and integrity, such that even a single base mutation may confuse the *DROSHA*, *DICER*, or AGO [97]. The possible dual strand hindrance by premature miRNA intervention may somehow jeopardize scientific rigor to study either of the sister strands, which creates rising concerns toward its future applications. However, from a different perspective, we see the potential of functionally synergistic sister strands in therapeutic intervention. If the miRNA precursor is present in the upstream of synergetic dual sister strands, then by targeting a single precursor, the modulation of two sets of genes can be achieved by simultaneously altered expressions of two sister miRNAs. Therefore, once disease-related sister miRNAs are established, targeting the premature miRNAs may serve as a therapeutic “one-two punch”.

## 6. Functional Annotation of Premature miRNAs in Cancers

**Figure 2:**
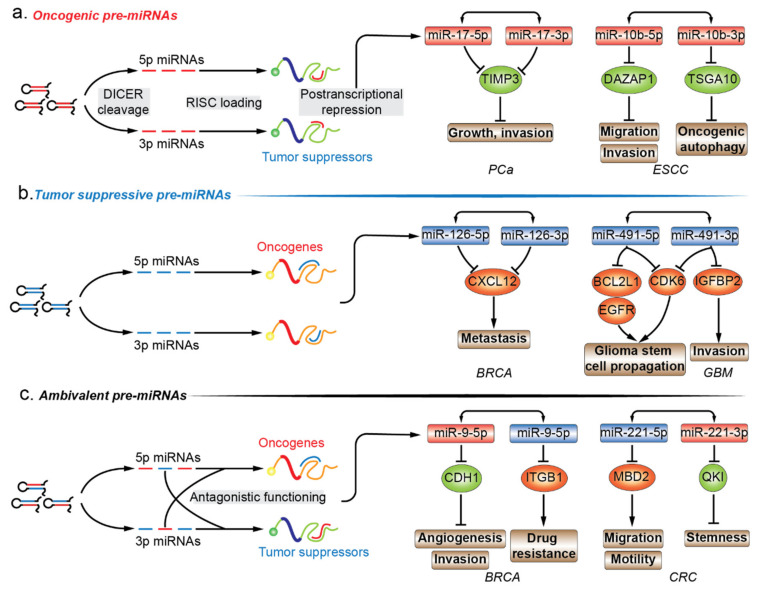
Functional annotation of miRNA precursors by dual regulatory strands. (**a**) The oncogenic pre-miRNAs produce two oncomiRs synergistically contributing to the oncogenic process under a certain cellular context by post-transcriptional regulation of tumor suppressors. (**b**) Conversely, the two mature products of tumor suppressive pre-miRNAs target two sets of oncogenes, respectively. (**c**) An ambivalent pre-miRNA is processed into two mature miRNAs that have simultaneous conflicting reactions toward cancer progressions. PCa, prostate cancer; ESCC, esophageal squamous cell carcinoma; BRCA, breast cancer; GBM, glioblastoma; CRC, colorectal cancer.

The functional importance of miRNAs is being extensively studied. The functional role of miRNA* species in various diseases is highly valuable in annotating the functionality of miRNA precursors [61]. Almost all models of functional miRNAs, from diverse origins and biosynthesis pathways, converge at the step where a hairpin miRNA precursor is produced, and thus, functional annotation of premature miRNA is focused on the last premature form of miRNA, the pre-miRNA. Given the inability to establish direct contact with regulatory protein complexes, the functions of pre-miRNAs are determined by the joint activity of their encompassing regulatory miRNAs. In this review, we have mainly focused on the pre-miRNAs that produce two mature strands and account for the majority of miRNA precursors. Mining functional information related to miRNA in combination with newly discovered biological implication of its passenger strand is essential for drafting the biological propensity of their parental precursor transcripts. In cancers, pre-miRNAs can be divided into three different subgroups, i.e., oncogenic, tumor suppressive, and ambivalent pre-miRNAs, based on the function of the dual sister strands (Figure 2a–c.).

### 6.1. Oncogenic Pre-miRNAs 

Similar to the definition of oncogenic miRNAs (oncomiRs), an oncogenic pre-miRNA produces two oncomiRs, which synergistically contribute to the oncogenic process under a certain cellular context (Figure 2a.; Table 2). For instance, Yang and his colleagues have shown that pre-miR-17 induces more malignant phenotypes in prostate cancer (PCa) and hepatocellular carcinoma (HCC) [72,73]. In PCa, luciferase reporter assay demonstrated that both miR-17-5p/3p targeted the 3’-UTR of TIMP metallopeptidase inhibitor 3 (*TIMP3*), and ectopic expression of pre-miR-17 promoted tumor growth and invasion, which was mediated by TIMP3 repression [72]. Additionally, pre-miR-17 transgenic mice were prone to develop HCCs. Unlike targeting a common tumor suppressor to augment the regulatory activity in PCa, pre-miR-17 displayed a different mode of action by targeting multiple tumor suppressive pathways in HCC. MiR-17-5p was bound to two putative binding sites of *PTEN*, while GalNAc transferase 7 (*GalNT*7) and *VIM* (namely *vimentin*) were regulated by the passenger strand miR-17-3p; all these regulatory activities are ceRNA-independent. The combined effect of miR-17-5p/3p led to a proliferative and migratory phenotype [73]. Additional examples include oncogenic pre-miR-10b in esophageal squamous cell carcinoma (ESCC) [74,75], pre-miR-21 in ovarian cancer [98,99,100], and pre-miR-221 in PCa [76,77], the dual tumor-promoting strands in all these cases have been identified by individual studies. First, starvation-induced miR-10b-5p expression increased the autophagic flux in ESCC during nutrient deprivation, and DAZ associated protein 1 (*DAZAP1*) was the direct target and mediator of this phenomenon. Decreased *DAZAP1*, an oncogenic signature, was observed in ESCC tumor samples and was correlated with poor survival, and this enhanced the migratory and invasive capacities in ESCC cell lines [74]. MiR-10b-3p expression increased under hypoxic conditions in ESCC cells, and its downregulation, mediated by hypoxia, impaired its migratory and invasive capabilities. This could be because the hypoxia-inducing miR-10b-3p suppressed the expression of targeting testis specific 10 (*TSGA10*), which contains a putative binding site at the 3’-UTR [75]. Second, miR-21-5p is located within chromosomal region of 17q23-25 and its amplification is correlated with shorter progression-free and overall survival in ovarian clear cell carcinoma (OCCC), one of the most lethal types of gynecological malignancies. miR-21-5p amplification demonstrated that tumor suppressor *PTEN* was a direct target of miR-21-5p, but miR-21-5p antagonization was not able to compromise cell proliferation or invasion of RMG-II cells, which is a newly established OCCC cell line [98]. However, in OVCAR-3 cells (low grade serous ovarian papillary adenocarcinoma), miR-21-5p knockdown increased in *PTEN* upregulation and decreased cell proliferation, migration, and invasion [99]. MiR-21-3p was identified as a potential miRNA mediator for the development of cisplatin resistance through microarray analysis performed between A2780 cell line and its cisplatin-resistant derivative, CP70. The acquired cisplatin resistance of A2780 could be induced by miR-21-3p overexpression, which can be witnessed in multiple ovarian cancer cell lines. In response to miR-21-3p upregulation, the decrease of its direct target, the neuron navigator 3 (*NAV3*), desensitized ovarian cancer cells to cisplatin [100]. Unexpectedly in the same paper, the introduction of miR-21-5p mimics sensitized A2780 cells (human ovarian cancer cell line) to cisplatin, and the underlying mechanism was not further investigated in the study [100]. The unusual anticancer effect of miR-21-5p contradicts the general notion of its canonical oncogenic role in various cancers, including ovarian cancers. By searching related terms in PubMed, we found several studies reporting the relationship between miR-21-5p and cisplatin resistance in ovarian cancer, and all the studies demonstrate that miR-21 contributes to the development of cisplatin resistant phenotype [101,102,103,104]. It is of particular interest to us as three out of four studies are performed in A2780 cell line or its variants as well, which indicates a similar cellular background. However, even the same experimental cell line in a different lab can introduce bias in phenotypic performance. Therefore, it is controversial in the function role of miR-21-5p in cisplatin resistance of ovarian cancer, and requires further validation in the same experimental model and at the same time. Lastly, a study showed that pre-miR-221 mediates the malignant transition in PCa. MiR-221-5p expression was elevated in tumor and adjacent tissues of clinical samples, indicating its oncogenic role in prostate carcinogenesis. Ectopic miR-221-5p manipulation proved that miR-221-5p enhanced the migratory and proliferative capabilities of PCa cells in vitro, and suppressers of cytokine signaling 1 (*SOCS1*), an established tumor suppressor, was validated to be the direct target of 3’-UTR luciferase reporter assay [76]. MiR-221-3p is reported to participate in the development or maintenance of castration resistant prostate cancer (CRPC) phenotype, but the underlying mechanism remains unknown [105]. Another study has demonstrated that ectopic upregulation of miR-221-3p promoted androgen independent cell growth in LNCaP cell lines. Two potential tumor suppressive targets, HECT domain E3 ubiquitin protein ligase 2 (*HECTD2*) and *RAB1A*, were identified by the combination of RNA immunoprecipitation and miR-221-3p target prediction programs. Reduction of *HECTD2* and *RAB1A* or increased miR-221-3p contributed to the development of CRPC phenotype [77]. Examples of oncogenic pre-miRNAs elucidate the preliminary therapeutic potential of dual oncomiRs ablation using only one pre-miRNA intervention.

### 6.2. Tumor-Suppressive Pre-miRNAs 

In contrast, a tumor suppressive pre-miRNA can be processed into dual tumor suppressive mature miRNAs, which simultaneously target two sets of oncogenes (Figure 2b.; Table 2). Tumor suppressive pre-miR-126 in breast cancer (BRCA) [78], pre-miR-144 in lung squamous cell carcinoma (LUSC) [79], pre-miR-524 and pre-miR-491 in glioblastoma (GBM) [80,81] are discussed in this section. To start with, miR-126-5p/3p were downregulated in metastatic BRCA cell lines and clinical samples, and ectopic pri-miR126 expression significantly decreased lung metastasis, implying the negative regulatory potential in cancer metastasis. With focus on cytokines/chemokines, *CXCL12* (also known as *Sdf-1α*) was downregulated in response to forced pri-miR-126 expression. The luciferase reporter assay using *CXCL12* 3′-UTR construct confirmed that either pri-miR-126 or its mature products, miR-126-5p/3p, suppressed the luciferase activity in BRCA cells. The suppression of the migratory capabilities of mesenchymal stem cells were mediated by downregulation of *CXCL12* through miR-126-5p/3p, thereby preventing the development of the metastatic phenotype [78]. Secondly, miR-144-5p/3p were downregulated in LUSQ tissues and cell lines, and high miR-144-5p/3p expression was correlated with a favorable outcome for LUSQ patient data in The Cancer Genome Atlas (TCGA) database. Transient miR-144-5p/3p transfection impaired the proliferative, invasive, and migratory abilities of LUSQ cells. Both miR-144-5p/3p were functional substrates of AGO-RISC, and neuronal calcium sensor 1 (*NCS1*) was a shared target for both strands. The phenotypic influence of neuronal calcium sensor 1 knockdown mimicked the counterpart of miR-144-5p/3p introduction [79]. Thirdly, miR-524-5p/3p were exclusively downregulated in the classical subtype of GBM and negatively associated with epidermal growth factor receptor (*EGFR*) expression, while *EGFR* amplification was observed in 97% of the classical subtypes and was infrequent in other subtypes [80,106]. EGFR could recruit the repressive histone modifier to the promoter region of *MIR524* and reduced pri-miR-524 production. TEA domain transcription factor 1 (*TEAD1*) and *SMAD2* were validated to be the target of miR-524-5p and -3p, respectively, and *HES1* was the target of both the strands. All three targets of pre-miR-524 converge on the activation of the downstream oncogene, *MYC*, resulting in retention of the migratory, invasive, and proliferative phenotypes [80]. Lastly, *MIR491* was found to be frequently codeleted with cyclin dependent kinase inhibitor 2A (*CDKN2A*) located on chromosome 9p21.3 in GBMs, and accordingly, the corresponding miR-491-5p and -3p expression levels were significantly lowered in GBM compared to in the normal brain. Reporter assays confirmed the binding sites within *EGFR* and BCL2 like 1 (*BCL2L1*) for miR-491-5p, insulin like growth factor binding protein 2 (*IGFBP2*) for miR-491-3p, and cyclin dependent kinase 6 (*CDK6*) for both. The concurrent loss of tumor suppressive miR-491-5p and -3p derepressed multiple oncogenes, consequently exacerbating malignancy in GBM in vitro and in vivo [81]. Based on the coordinated effect of the two mature products of pre-miRNA on cancer suppression, supplementation with two tumor suppressors through introduction of a single miRNA precursor might promisingly outweigh a single miRNA amendment for future therapeutic purposes.

### 6.3. Ambivalent Pre-miRNAs 

Unlike the aforementioned oncogenic or tumor suppressive pre-miRNA, ambivalent pre-miRNAs can be processed into two mature miRNAs which tend to function in two opposing directions (Figure 2c; Table 2). For example, pre-miR-31 in oral squamous cell carcinoma (OSCC), pre-miR-9 and pre-miR-10b in BRCA consist of a single oncomiRs at the 5′ arm and tumor suppressive miRNA at the 3′ arm, while pre-miR-221 in colorectal cancer (CRC) are constituted in a reverse manner [82,83,84,85,86,87,88,89]. With respect to pre-miR-31, the oncogenic miR-31-5p in head and neck squamous cell carcinoma (HNSCC) were found to be one of the most highly expressed miRNAs when compared to normal tissues. The elevated miR-31-5p expression was linked to increased oncogenic potential of HNSCC cells by promoting cell growth and migration. The phenotypic effect of miR-31-5p was mediated by the suppression of factor-inhibiting hypoxia-inducible factor (*FIH*), which is the direct target of miR-31-5p. However, under normoxic conditions, *FIH* acts as a tumor suppressor by inhibiting oncogenicity [82]. The tumor suppressive complementary strand, miR-31-3p, was associated with decreased capability of growth and migration in SAS and Fadu OSCC cell lines. Ras homolog family member A (*RHOA*) was chosen as a candidate for observing the phenotypic changes in response to miR-31-3p manipulation and the miRNA-target relationship was verified by 3′-UTR luciferase reporter assays. Interestingly, the authors examined the phenotypic influence of forced pre-miR-31 expression, which supplemented two mature products in the form of oncogenic 5′ arm and tumor suppressive 3′ arm, and showed that the phenotypic profiling of ambivalent pre-miR-31 introduction was oncogenic [83]. Regarding pre-miR-9, oncogenic miR-9-5p was found to be significantly upregulated in human BRCA compared to normal breast tissues. Besides, c-myc induced an increase of more than 500-fold in the expression of miR-9-5p/3p in mice transgenic models [107,108]. In human cancer, miR-9-5p displayed a positive correlation with MYCN amplification and tumor grade neuroblastoma, and was elevated in patients with metastatic BRCA. In BRCA cells, 3′-UTR of cadherin 1 (*CDH1*) was directly targeted by miR-9-5p. CDH1 suppression derepressed β-catenin pathways and induced vascular endothelial growth factor A (*VEGFA*) expression levels, angiogenesis, mesenchymal transitions, and metastatic phenotypes in BRCA [84]. The pairing strand, viz., tumor suppressive miR-9-3p, was screened out from a miRNA mimics library for a possible synthetic sensitizer of *MEK* inhibitor, viz., AZD6244 in BRCA cell lines. In the presence of miR-9-3p, AZD6244-treated BRCA cells were subjected to vulnerable growth arrest, migration, and inhibition of invasion, which was mediated by miR-9-3p repression of integrin subunit beta 1 (*ITGB1*) [85]. With regards to pre-miR-10b, oncogenic miR-10b-5p was highly expressed in metastatic BRCA, and its upregulation in BRCA cell lines was competent enough to increase the cell motility and invasiveness in vitro and elicit tumor invasion and distant metastasis in vivo. The increase of miR-10b-5p was induced by *TWIST1*-binding to E-box located at the upstream of *MIR10b* stem-loop sequences, and the function was executed by direct targeting of 3′-UTR of homeoboxD10 (*HOXD10*). This is an established tumor suppressor system which impairs the migrating and invading abilities of BRCA cells in vivo [86,109]. The decreased miR-10b-3p expression was identified in BRCA tumor samples compared with matched peritumor tissue samples, indicating that the loss-of-function may contribute to tumorigenesis. In BRCA cell lines, ectopic introduction of miR-10b-3p inhibited cell viability in vitro and tumor growth in xenograft models. The possible molecular mechanism is the direct repression of the regulatory genes of cell cycle, viz., cyclin A2 (*CCNA2*), polo like kinase 1 (*PLK1*) and *BUB1*, whose expression levels were the most negatively correlated with miR-10b-3p in BRCA samples. The restoration of miR-10b-3p in BRCA cell lines increased subG1- and decreased S-phase cell populations by inhibiting dysregulated cell cycle-associated pathways, and all the three validated targets were associated with poor outcome in BRCA patients [87]. In contrast to the above-mentioned oncogenic 5′ arm-derived miRNA, tumor suppressive miR-221-5p were significantly downregulated in metastatic CRC cells, and its downregulation was associated with poor survival of CRC patients belonging to all the stages. Upregulation of miR-221-5p reduced the subcutaneous and orthotopic CRC tumor volumes in mice, and impaired migratory and invasive capability of CRC cells in vitro. Methyl-CpG binding domain protein 2 (*MBD2*) contains a validated binding site for miR-221-5p, and its knockdown recaptured the phenotypic change of miR-221-5p introduction in CRC cells [88]. The oncogenic strand of pre-miR-221 in human CRC, miR-221-3p, was preferentially overexpressed in CRC stem cell-like cells, and its overexpression correlated with the reduced survivability in CRC patients. Overexpressed miR-221-3p promoted the three-dimensional (3D) organoid-forming capacity of human CRC cells in vitro, and miR-221-3p antagonization repressed in vivo engraftment and growth of human CRC patient-derived xenografts (PDX) in mice models. *QKI-5* was experimentally validated to be the functional target of miR-221-3p, which impeded the 3D-organoid formation and tumorigenic capability of human CRC PDX cells [89]. In certain cellular backgrounds, therapeutic manipulation of ambivalent pre-miRNAs may be infeasible as the expression of the dual sister strands are inevitably interfered simultaneously and unidirectionally, which leads to transcriptional repression of oncogenes as well as tumor suppressors.

## 7. The “Ongoing” Premature miRNA Interventions

The widespread biological implication of miRNAs has been recognized and accepted, and a variety of functional miRNAs have been proposed for therapeutic, diagnostic, or prognostic purposes. It has been reported that the miRNA expression profiles can create unique signatures between different cancer types, which may rely on the tissue-dependent expressions and strand-selections of miRNAs [63,110,111]. The miRNA-based therapeutics depend on the depletion of oncomiRs and the supplementation of tumor suppressive miRNAs. Strategies based on modified oligonucleotides, expression cassettes, and small-molecule drugs are promisingly developed to target certain steps of miRNA biogenesis and functioning machinery with high specificity and low toxicity, which has been comprehensively reviewed by our team in [91]. Embracing the sanative expectation, some of the miRNA therapies are trialed for clinical values (Table 3) [112,113,114]. The therapeutic potential of mature miRNA paves the way for targeting synergistic pre-miRNAs, and fortuitously, some of the miRNA-targeting strategies are executed by manipulating premature miRNAs. The insertion of a pre-miRNA or pri-miRNA-expressing cassettes into commercially available vectors, like plasmids and viral vectors, are widely used to overexpress targeted miRNAs. Such methods may introduce unexpected bias for interpreting the phenotypic change correlated with certain miRNAs; however, from a different angle, this is a valid strategy for premature miRNA intervention. Synthetic pre-miRNA or pri-miRNA are reported, and are able to incorporate into miRNA processing pathways, producing two functional mature products from both arms [115,116]. For instance, synthetic pre-miR-34a is a chemically synthesized pre-miRNA with complete sequence identity and can be easily transfected into cells. The suppression of sirtuin 1 (*SIRT1*) and *SP4* by miR-34a-5p and tumor necrosis factor (*TNF*) by miR-34a-3p could be reached by the introduction of synthetic pre-miR-34a. This study not only proves the dual strand activity of pre-miR-34a but also provides an ideal and affordable platform for further investigation. Small molecule drugs comprise the vast majority of approved drugs, and in miRNA biology, some functional small molecules, such as Benzimidazole (pre-miR-96 inhibitor) [117], Azobenzene 2 (pri-miR21 inhibitor) [118], AC1MMYR2 (pre-miR-21 inhibitor) [119], and Enoxacin (*TRBP* activator) [120], are designated to interfere with the miRNA biogenesis with therapeutic potential. *TRBP*, a key component of *DICER*-processing complex, is the target protein of a small molecule activator, Enoxacin. Increase in the production of miRNAs triggered by Enoxacin are universal and lack selectivity [120]. However, the other three small molecule inhibitors of miRNAs are designed with unique selectivity. Benzimidazole and AC1MMYR2 are two small molecule inhibitors specifically targeting pre-miR-96 and pre-miR-21, respectively. Though using different computational methods, the simulation of RNA motif–small molecule interactions provides insight into the underlying screen of lead compounds from a library of small molecules consisting of thousands of compounds. Preoccupation of pre-miRNA by small molecules prohibit *DICER*-processing and consequently reduces the production of target miRNA. These two compounds were tested with high bioactivity and selectivity [118,119]. Regardless of pre-miRNA secondary structure, another compound-screening method relies on the high-throughput luciferase reporter assay, and experimental quantification of luciferase signal of multiple reporters can reveal the selectivity and bioactivity of certain compounds. Azobenzene 2 was found to selectively affect the expression levels of primary transcript and precursor of miR-21-5p other than that of miR-30a-5p, but the exact mechanism requires further investigation [118]. It is a pity that all these small molecules are tested only for efficacy on the guide strand, whereas the undetermined star strand should simultaneously be affected by the varying availability of premature miRNAs. Recently, editing the gene of miRNA has received unprecedented attention, because the development of clustered regularly interspaced short palindromic repeats (CRISPR) technology has democratized and facilitated widespread genome engineering. CRISPR and CRISPR-associated (Cas) proteins creates an easy-to-use and state-of-the-art system for researchers to perform genome editing, base editing, and gene transcriptional regulation in living cells and organisms [121]. The genomic editing of miRNAs alters the production of miRNA primary transcripts, which is the premature form of miRNA product. Given the emerging role of CRISPR-Cas system in miRNA editing in cancer research, it is predictable that this strategy holds tremendous promise for future therapeutic interventions using premature miRNAs as targets [122]. Collectively, in light of the current methodology of mature miRNA manipulations, interventions using premature miRNAs are already ongoing, but its overall consequence and therapeutic superiority has not yet been fully recognized.

## 8. Conclusion and Future Perspective

Without any doubt, miRNA species have grown to be one of the most important regulatory molecules in maintaining intracellular hemostasis. The evolving knowledge about the biogenesis and function of miRNAs keeps illuminating miRNA biology through unprecedented depth and breadth. The early stage of functional evaluation of miRNA species is focused mostly on the guide strands of miRNA duplex, and the passenger strand is considered to be nonsense and degraded after the guide strand has been loaded into the RISC. With the increase in evidence concerning substantial regulatory activities of miRNA*, the nonsense tag of passenger strand has been gradually torn off. As a consequence, some of the currently available mature miRNA-targeting strategies have been deemed less rigorous in cancer research due to the influence of nonvoluntary negligence in miRNA* expressions. Reviewing the various biogenesis pathways leading to functional miRNAs, impertinence with respect to these inappropriately applied strategies converge on the attempt to manipulate a single miRNA at premature stages. This should be avoided such that simultaneously altered dual strands could either mitigate or exaggerate the performance of the target miRNA-mediated phenotype. One of the major advantages of premature miRNA manipulation rises from the lesson of utilizing such methodology in single miRNA manipulation. If the dual sister strands function synergistically, it would not be more appropriate to use the same strategies for exaggerating the therapeutic performance. The theory of “target-two-sets-of-genes-with-one-pre-miRNA” has been proposed. Currently, only a very small portion of studies are dedicated to establishing the functional relevance of dual sister strands in one study. Functional annotation of a pre-miRNA based on multiple separated studies might face a conflicted situation that ambivalent functions of one of the mature strands are reported. A problem is that the investigations of dual sister strands of a miRNA precursor are mostly not launched in the same lab at the same period of time. It is inevitable that a contradicted phenotype occurs due to the different experimental conditions. However, it can be anticipated that in company with the developing understanding of miRNA* species, functional annotation of pre-miRNA will be taken into account, and annotated pre-miRNA-based therapeutic will sprout with less controversy. Theoretically, a pre-miRNA should be functionally annotated in a single study to rule out the experimental bias, and ideally, phenotypical profiling should be performed before therapeutic interventions. The phenotypic profiling of dual regulatory strands may grant superior performance to the therapeutic interventions on certain premature miRNA, which acts as a “one-two punch” strategy against malignancies. The functional annotation of a miRNA precursor will be generously aided by the availability of annotated miRNA species. We envision that in the upcoming future, there will be a significant decrease in the inappropriate utilization of premature miRNA inventions on single miRNA manipulations and generous increase in the number of functional miRNA precursor annotations. Being an almost indispensable step of miRNA maturation, the miRNA precursor may deserve a functional tag in the future, acting as a molecular target instead of a mere carrier of regulatory miRNA/miRNA*.

## Figures and Tables

**Table 1:** Selected examples of non-mRNA–miRNA interactions.

RNA Species	Decoy RNAs	Target miRNA(s)	Ref.
Pseudogenes	PTENP1	miR-20a, miR-19b, miR-21, miR-26a, and miR-214	[48]
	TUSC2P	miR-17, miR-93, miR-299-3p, miR-520a, miR-608, and miR-661	[49]
LincRNAs	LincRNA-p21	let-7	[50]
	Linc-RoR	miR-145-5p, miR-181a-5p, and miR-99b-3p	[51]
	Linc00673 mutant	miR-1231	[52]
T-UCRs	Uc.160	miR-155, miR-24	[53]
	Uc.346A	miR-155	
	Uc.348	miR-29b	
	Uc.339	miR-339-3p, miR-663b-3p, and miR-95-5p	[54]
CircRNAs	ciRS-7	miR-7	[55]
	Sry	miR-138	
	CDR1as	miR-7	[56]

Abbreviation: long intergenic noncoding RNAs (LincRNAs); Transcribed Ultraconserved Regions (T-UCRs); circular RNAs (CircRNAs).

**Table 2:** Selected examples of functionally annotated pre-miRNAs with dual regulatory strands.

Annotation	Mature Products	Previous ID	Clustered miRNAs	Type of Cancer	Targets	Biological Implications	Ref.
***Oncogenic pre-miRNAs***	miR-17-5p/3p	miR-17/miR-17 *	miR-17~92	Prostate cancer	*TIMP3*	Growth↑, invasion↑	[72]
				Hepatocellular carcinoma	*PTEN, GalNT7, VIM*	Proliferation↑, migration↑	[73]
	miR-10b-5p/3p	miR-10b/miR-10b *	_	Esophageal squamous cell carcinoma	*DAZAP1, TSGA10*	Oncogenic autophagy↑, growth↑, metastasis↑	[74,75]
	miR-221-5p/3p	miR-221 */miR-221	miR-222/221	Prostate cancer	*SOCS1, HECTD2, RAB1A*	Proliferation↑, migration↑, androgen independent growth↑	[76,77]
***Tumor-suppressive pre-miRNAs***	miR-126-5p/3p	miR-126 */miR-126	_	Breast cancer	*CXCL12*	Metastasis↓	[78]
	miR-144-5p/3p	miR-144 */miR-144	miR-4732~451a	Lung squamous cell carcinoma	*NCS1*	Proliferation↓, invasion↓, migration↓	[79]
	miR-524-5p/3p	miR-524 */miR-524	miR-520b~520d	Glioblastoma	*TEAD1, SMAD2, HES1*	Migration↓, proliferation↓	[80]
	miR-491-5p/3p	miR-491/-	_	Glioblastoma	*BCL2L1, EGFR, CDK6, IGFBP2*	Proliferation↓, invasion↓, glioma stem cell propagation↓	[81]
***Ambivalent pre-miRNAs***	miR-31-5p/3p	miR-31/miR-31 *	_	Oral squamous cell carcinoma	*FIH, RHOA*	Viability↑, migration↑↓, growth↓	[82,83]
	miR-9-5p/3p	miR-9/miR-9 *	_	Breast cancer	*CDH1, ITGB1*	Angiogenesis↑, motility↑, invasion↑, drug resistance↓	[84,85]
	miR-10b-5p/3p	miR-10b/miR-10b *	_	Breast cancer	*HOXD10, BUB1, PLK1, CCNA2*	Metastasis↑, cell cycle regulation↓, proliferation↓	[86,87]
	miR-221-5p/3p	miR-221*/miR-221	miR-222/221	Colorectal cancer	*MBD2, QKI*	Migration↓, motility↓, stemness↑	[88,89]

The genes in red/blue indicate the implications in the pro/antioncogenic processes; Abbreviations: TIMP metallopeptidase inhibitor 3 (*TIMP3*); phosphatase and tensin homolog (*PTEN*); GalNAc transferase 7 (*GalNT7*); vimentin (*VIM*); DAZ associated protein 1 (*DAZAP1*); targeting testis specific 10 (*TSGA10*); suppressers of cytokine signaling 1 (*SOCS1*); HECT domain E3 ubiquitin protein ligase 2 (*HECTD2*); neuronal calcium sensor 1 (*NCS1*); TEA domain transcription factor 1 (*TEAD1*); BCL2 like 1 (*BCL2L1*); epidermal growth factor receptor (*EGFR*); cyclin dependent kinase 6 (*CDK6*); insulin like growth factor binding protein 2 (*IGFBP2*); factor-inhibiting hypoxia-inducible factor (*FIH*); Ras homolog family member A (*RHOA*); cadherin 1 (*CDH1*); integrin subunit beta 1 (*ITGB1*); homeoboxD10 (*HOXD10*); cyclin A2 (*CCNA2*); polo like kinase 1 (*PLK1*); methyl-CpG binding domain protein 2 (*MBD2*).

**Table 3:** Selected examples of developing miRNA therapeutics.

Intervention/Treatment (Company)	Target miRNA	Oligonucleotide Format	Condition(s) or Disease(s)	Stage	Clinical Trials.gov Identifier:	Ref.
Mature miRNA-based strategies						
Miravirsen (Santaris Pharma A/S)	miR-122	Locked Nucleic Acid-modified DNA phosphorothioate antisense oligonucleotide	Hepatitis C	Phase 2a	NCT01200420	[112]
MRX34 (Mirna Therapeutics, Inc.)	miR-34a	Liposomal mimic	Primary liver cancer Lymphoma Melanoma Multiple myeloma Renal cell carcinoma Lung cancer	Phase 1	NCT01829971	[113]
TargomiRs (EnGeneIC Limited)	miR-16	Minicells (EnGeneIC Dream Vectors) loaded with mimic microRNA	Malignant pleural mesothelioma Nonsmall cell lung cancer	Phase 1	NCT02369198	[114]
